# A Randomized, Triple‐Blind, Placebo‐Controlled, Parallel Clinical Trial Investigating Safety and Efficacy of Corn Leaf Extract on Sleep Quality in a Healthy Population With Difficulty Falling or Staying Asleep

**DOI:** 10.1002/fsn3.71285

**Published:** 2026-01-02

**Authors:** Katarina M. Doma, Abdelrahman Zamzam, David C. Crowley, Najla Guthrie, Erin D. Lewis

**Affiliations:** ^1^ KGK Science Inc. London Ontario Canada

**Keywords:** corn leaf extract, dietary supplement, EEG, sleep

## Abstract

Difficulties falling and/or staying asleep affect over one quarter of adults in the United States. Current management strategies include prescription sleep aids. However, long‐term use is associated with serious adverse effects. Therefore, natural alternative sleep aids that may provide safer and more effective relief of sleep disturbances are needed. In this randomized, triple‐blind, placebo‐controlled clinical trial, 80 healthy adults (*n* = 40 per group) with difficulties falling and/or staying asleep were supplemented with a standardized corn leaf extract (CLE) or placebo for 28 days. Objective (actigraphy with electroencephalogram) and subjective (Pittsburgh Sleep Quality Index) sleep measures, serum serotonin, plasma melatonin, and gamma‐aminobutyric acid were assessed at baseline (Day 0), Day 14, and Day 28, and safety was assessed at screening and Day 28. Compared to placebo, participants supplemented with CLE demonstrated statistically significant increases in total sleep time (TST) and light sleep at Day 28 and improvements in REM sleep at both Days 14 and 28. Further, participants supplemented with CLE had significantly fewer sleep interruptions and shorter sleep onset latency at Day 14 with shorter wake after sleep onset (WASO) and higher sleep efficiency at Days 14 and 28. Post hoc analysis supported these findings with a significant increase of 35.7 min in non‐REM sleep at Day 28 for participants supplemented with CLE compared to a decrease of 10.6 min for those on placebo. Supplementation with CLE was safe and well tolerated. Findings suggest CLE supplementation may improve sleep parameters in a healthy population with sleep difficulties.

## Graphical abstract

Corn leaf extract (CLE) contains a naturally occurring substance, 6‐methoxybenzoxazolinone (6‐MBOA). Structurally similar to melatonin, 6‐MBOA acts as a weak β‐adrenergic agonist with an affinity for melatonin receptors and stimulating melatonin synthesis. In a randomized, placebo‐controlled clinical trial of individuals with difficulty falling and/or staying asleep, CLE supplementation resulted in a significant increase in total, REM, and light sleep time, in comparison to placebo, as well as a reduction in sleep interruptions and shorter sleep onset latency.

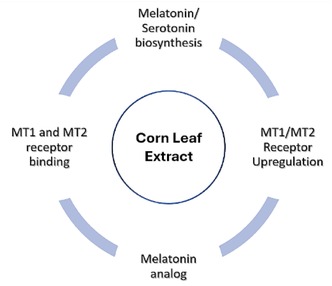

## 1 Introduction

Sleep is an essential physiological phenomenon that has a cyclical impact on basic human functioning, both physically and psychologically (Salehinejad et al. 2022). According to a data brief by the National Center for Health Statistics (NCHS), in 2020, 14.5% and 17.8% of adults in the U.S. had trouble falling asleep and staying asleep, respectively, most days or every day for the past 30 days (NCHS 2022). Additionally, Hou et al. (2023) reported that an estimated 27% of U.S. adults self‐reported experiencing sleep disturbances and approximately 6.3% of adults in the U.S. took sleep medication every day in the last 30 days (NCHS 2023). Data suggest that prescription sleep aid use is more prevalent among older adults, with 11% of those aged 65 and overreporting use (NCHS 2023). However, long‐term use of these therapies, such as benzodiazepine receptor agonists, sedating antidepressants, and antiepileptics, is associated with serious adverse effects (Sleep Foundation 2020). Long‐term users may experience confusion, diarrhea, dizziness, memory impairment, and weakness (Sleep Foundation 2020). Natural, complementary, and alternative sleep aids may provide safer and more effective relief of sleep disturbances in healthy populations.

Corn leaf extract (CLE) is a standardized ethanol extract derived from the young leaves of *Zea mays* L., or corn, and contains a naturally occurring substance, 6‐methoxybenzoxazolinone (6‐MBOA). Structurally similar to melatonin, 6‐MBOA acts as a weak β‐adrenergic agonist with an affinity for melatonin receptors, resulting in melatonin synthesis (Anderson et al. 1988; Sweat and Berger 1988; Yuwiler and Winters 1985). Melatonin promotes sleep through the activation of high‐affinity, G protein‐coupled receptors, referred to as MT1 and MT2 (Gobbi and Comai 2019). The MT1 receptor is mainly associated with the regulation of rapid eye movement (REM) sleep, whereas MT2 regulates non‐REM sleep (NREM) (Gobbi and Comai 2019). In a recent pre‐clinical study, CLE was tested on freely moving rats equipped with a surgically installed electroencephalography (EEG) device, which showed a statistically significant increase in TST, NREM, and SWS (deep sleep) while decreasing awake time (Kim et al. 2024). CLE has also been previously found to improve mood in individuals with mild to moderate anxiety and depression (Kim et al. 2024; Talbott et al. 2023). Further, CLE supplementation has not been associated with serious adverse events or clinically relevant changes in vital signs (Kim et al. 2024; Talbott et al. 2023). Therefore, the objective of the current study was to investigate the efficacy and safety of CLE on sleep quality in a healthy population with difficulty falling asleep or staying asleep.

## 2 Materials and Methods

### 2.1 Study Design and Approvals

**FIGURE 1:**
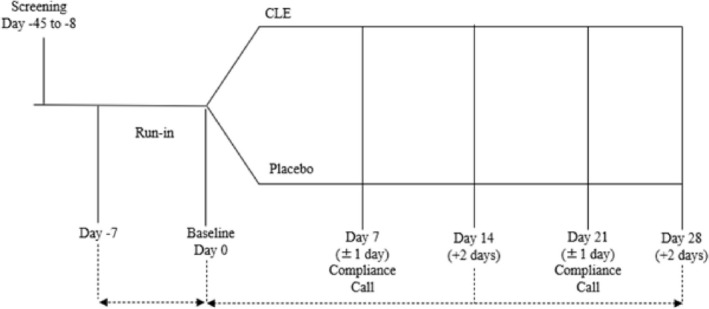
Study design.

This randomized, triple‐blind, placebo‐controlled clinical trial consisted of a 7‐day run‐in period followed by a 28‐day study period with efficacy outcome assessments conducted at baseline (Day 0), Day 14, and Day 28, and safety assessed at screening and Day 28 (Figure 1). The study was conducted at KGK Science Inc. (London, Ontario, Canada) from January 14, 2023, to November 30, 2023. Notice of authorization was granted on October 31, 2022, by the Natural and Non‐Prescription Health Product Directorate, Health Canada, and unconditional approval was granted on November 18, 2022, by the Institutional Review Board (Advarra, Aurora, Ontario, Canada; Pro00067855). This study was conducted in accordance with the ethical principles that originate in the Declaration of Helsinki and its subsequent amendments, reported according to the Consolidated Standards of Reporting Trials. The trial was retrospectively registered at the ISRCTN Registry (ISRCTN13394796) because of the confidential nature of the study design and investigational product.

### 2.2 Randomization and Blinding

The study products were identical in appearance and labeled per the requirements of ICH‐GCP and applicable local regulatory guidelines. Unblinded personnel at KGK Science Inc. who were not involved in study assessments labeled the study products. Investigators, other site personnel, and participants were blinded to the allocation of study products. A randomization list was generated using www.randomization.com, indicating the order of randomization, and was provided to the Qualified Investigator (QI). Block randomization was implemented to reduce bias and achieve balance in the allocation of participants to study groups.

### 2.3 Study Population

Participants were healthy adults aged 18–65 years, inclusive, with self‐reported difficulty falling asleep (taking longer than 30 min to fall asleep) or staying asleep, with two or more episodes of sleep disturbance (difficulty falling/staying asleep) in a 7‐day period for at least 1 month prior to baseline. Participants agreed to stay in the current time zone for the duration of the study period. Additionally, participants were instructed to refrain from herbal teas affecting sleep within 2 h of bedtime, unless currently part of their night routine for more than 30 days and were willing to maintain that routine, and from consuming caffeine and other stimulants (e.g., energy drinks) after 3:00 p.m. Further, participants agreed to maintain current lifestyle habits, including diet, medications, supplements, exercise, and avoidance of new supplements.

Exclusion criteria included individuals who were pregnant, breast feeding, or planning to become pregnant during the study period; allergy, sensitivity or intolerance to the ingredients in study products; previously diagnosed sleep disorder or use of a C‐PAP machine; menopausal women currently experiencing hot flashes; and any other condition or lifestyle factor the QI deemed may adversely affect the participant's ability to complete the study or its measures or pose significant risk to the participant.

Informed consent was obtained from all participants during the screening visit prior to performing study procedures.

### 2.4 Study Outcomes

The primary outcome was changes in objective sleep measures, including total sleep time, REM sleep time, deep sleep time, light sleep time, and awake duration from baseline at Day 28. Secondary outcomes included all objective sleep measures (total, REM, deep and light sleep, awake duration, sleep efficiency, sleep latency, number of awakenings, number of awakenings > 3 min), subjective sleep, and serotonin, melatonin, and gamma‐aminobutyric acid (GABA) from baseline at Days 14 and 28. Safety was assessed by adverse events, clinically relevant changes in vital signs, and clinical chemistry and hematology parameters.

### 2.5 Study Measures and Markers

Objective sleep measures were assessed by an actigraphy device with an EEG/electrooculography (EOG) combi sensor [SOMNOtouch RESP device (SOMNOmedics GmbH, Randersacker, Germany)]. The device is a portable, at‐home physiological signal recorder that records, monitors, displays, and stores data on biophysical sleep parameters, including body position and movement. Participants were instructed to wear the actigraphy and EEG/EOG electrodes during sleeping hours for seven nights prior to all study visits. To account for potential discomfort during initial use of the device, the first two nights of each seven‐night wearing period were deemed an acclimation period. If readings from the acclimation period were of sufficient quality to allow for automatic scoring and manual verification, they were permitted to be utilized in the analysis. Following automatic scoring of sleep reports through the SOMNOmedics software, manual verification of each report for participants who had at least four complete nights of sleep records prior to any study visit was completed by trained research personnel (Malhotra and Avidan 2013; Sleep Computing Committee & Japanese Society of Sleep Research 2012). The objective measure of NREM was computed as the sum of deep and light sleep.

Subjective sleep was assessed using the Pittsburgh Sleep Quality Index (PSQI) at all study visits (Buysse et al. 1989). Nineteen individual items generated seven “component” scores, including subjective sleep quality, sleep latency, sleep duration, habitual sleep efficiency, sleep disturbances, use of sleeping medication, and daytime dysfunction. A global score was calculated by summing the seven component scores, with a higher global score indicating worse sleep quality. The PSQI was purchased, and the contract was executed on October 31, 2022.

Blood was collected at each study visit by trained personnel for the analysis of serotonin (LifeLabs, London, Ontario, Canada) and melatonin and GABA (CannaLabs, London, Ontario, Canada). For the analysis of serotonin, the blood collection tube was inverted five times and left to clot at room temperature for 60 min, followed by centrifugation for 10 min at 3200 rpm at 25°C. The serum was aliquoted and stored at −80°C until analysis. Serotonin was analyzed using High Performance Liquid Chromatography with a lower limit of detection of 10 ng/mL. For the analysis of melatonin, the blood collection tube was centrifuged for 10 min at 1500 ***g*** at 4°C, and for GABA, the tube was centrifuged for 15 min at 1300 ***g*** at 4°C. Aliquots of plasma were stored at −80°C until analysis. Melatonin and GABA were analyzed using enzyme‐linked immunosorbent assays and the lower limits of detection were 6.88 pmol/L and 31.25 pg/mL, respectively.

Safety assessments included adverse events reported in study diaries and during study visits and were classified on the basis of the description, duration, intensity, frequency, and outcome. The QI determined the causal relationship of the investigational product to the AE as either “not related”, “unlikely”, “possible”, “probable”, or “most probable”. Vital signs, including blood pressure and heart rate, were measured with the participant seated comfortably with their back supported and the upper arm bared without restrictive clothing. Two readings were collected, and if there was a questionable disparity, a third reading was taken.

Laboratory safety markers included clinical chemistry [aspartate aminotransferase, alanine aminotransferase, alkaline phosphatase, total bilirubin, creatinine, electrolytes (sodium, potassium, and chloride), random glucose, and estimated glomerular filtration rate] and hematology parameters [white blood cell count with differential (neutrophils, lymphocytes, monocytes, eosinophils, and basophils), red blood cell (RBC) count, hemoglobin, hematocrit, platelet count, immature granulocytes, nucleated RBC, RBC indices (mean corpuscular volume, mean corpuscular hemoglobin, mean corpuscular hemoglobin concentration, and red cell distribution width)]. All blood safety markers were analyzed by LifeLabs using standardized procedures.

### 2.6 Intervention and Compliance

The investigational product (IP), CLE with trade name Maizinol (Lot FP220331‐01), is an ethanol (70%) extract of a non‐genetically modified corn, *Zea mays* , from fresh leaves harvested after 20 to 30 days of germination at about 30 cm height. After washing, the fresh corn leaves were fed into a cut machine to cut to size (about 2–3 cm). Extraction was carried out with about 15 times the volume of 70% ethanol at 80°C twice. Afterward, a 60°C vacuum was utilized to remove most of the ethanol. The concentrated extract was then spray‐dried at 140°C yielding dry CLE. The extraction ratio was 60:1 standardized to contain 0.2% to 0.3% (w/w) 6‐MBOA using pure 6‐MBOA standard (Aldrich 54,355–1) as a quantitative biomarker and measured using high‐performance liquid chromatography by Unigen Inc. (Tacoma, WA, USA). The IP contained 500 mg of corn leaf extract, gelatin, titanium dioxide, brilliant blue FCF sodium salt, and allura red AC, which met all specification parameters and complied with specified limits for heavy metals, pesticides, organic solvent residues, and microbial plate count according to USP guidelines. Other components in CLE included carbohydrates (~35%), protein (~25%), polyphenols, potassium (~8%), and calcium (~1.1%). The IP was stored in tightly closed containers at room temperature, away from heat, moisture, and light. The placebo contained microcrystalline cellulose, gelatin, titanium dioxide, brilliant blue FCF sodium salt, and allura red AC.

Study products were distributed to participants at baseline and Day 14, and participants were instructed to take one capsule per day, 60 min before bedtime, starting on Day 0. Participants were instructed to record missed doses in their study diary and continue regular supplementation the following evening. Compliance was calculated by dividing the number of study capsule units taken by the number expected to have been taken, multiplied by 100.

### 2.7 Statistical Analysis

The primary outcome of the study was a change in objective sleep measures, including total, REM, deep, and light sleep, as well as awake duration from baseline at Day 28. The planned sample size was 80 participants, with 40 participants assigned to each group. This sample size was determined to provide 80% power to detect differences in mean changes from baseline at Day 28 between the groups for the primary sleep measures. The calculation was based on a 5% significance level, accounted for 20% attrition, and used an effect size of 0.36 on the basis of Cohen's suggested effect sizes (Cohen 2013), supported by a previously published randomized controlled trial that objectively measured sleep outcomes (Kunz et al. 2004). The sample size was computed in G*Power 3.1.

Results are presented for the Intention‐to‐treat (ITT) population, which included all participants who received either product and had post‐randomization efficacy data. For demographics and general health data, descriptive statistics, including means, standard deviations, medians, and minimum and maximum values for continuous variables, and proportions for categorical variables, were calculated. The normality of continuous variables was assessed using the Shapiro–Wilk test. If normality was confirmed, group differences were analyzed using two‐sample t‐tests; otherwise, the Wilcoxon rank‐sum test was applied. Categorical variables were compared between groups using Chi‐square or Fisher's exact tests. Primary and secondary outcomes were summarized using means and standard deviations and were analyzed using linear mixed models. These models incorporated fixed effects for group, visit, and their interaction, with participant ID treated as a random effect. *P*‐values were reported, and model adequacy was evaluated using Q‐Q plots and plots of residuals versus fitted values. Cohen's d (*d*) effect sizes were also calculated where relevant. Post hoc analysis of NREM sleep time and specific sleep stages (1, 2, 3, 4) followed the same statistical approach as the primary outcomes analysis. The Statistician remained blinded for all planned and post hoc analyses. A significance level of *p*‐value ≤ 0.05 was used, and all analyses were performed using R 4.3.2 statistical software.

## 3 Results

**FIGURE 2:**
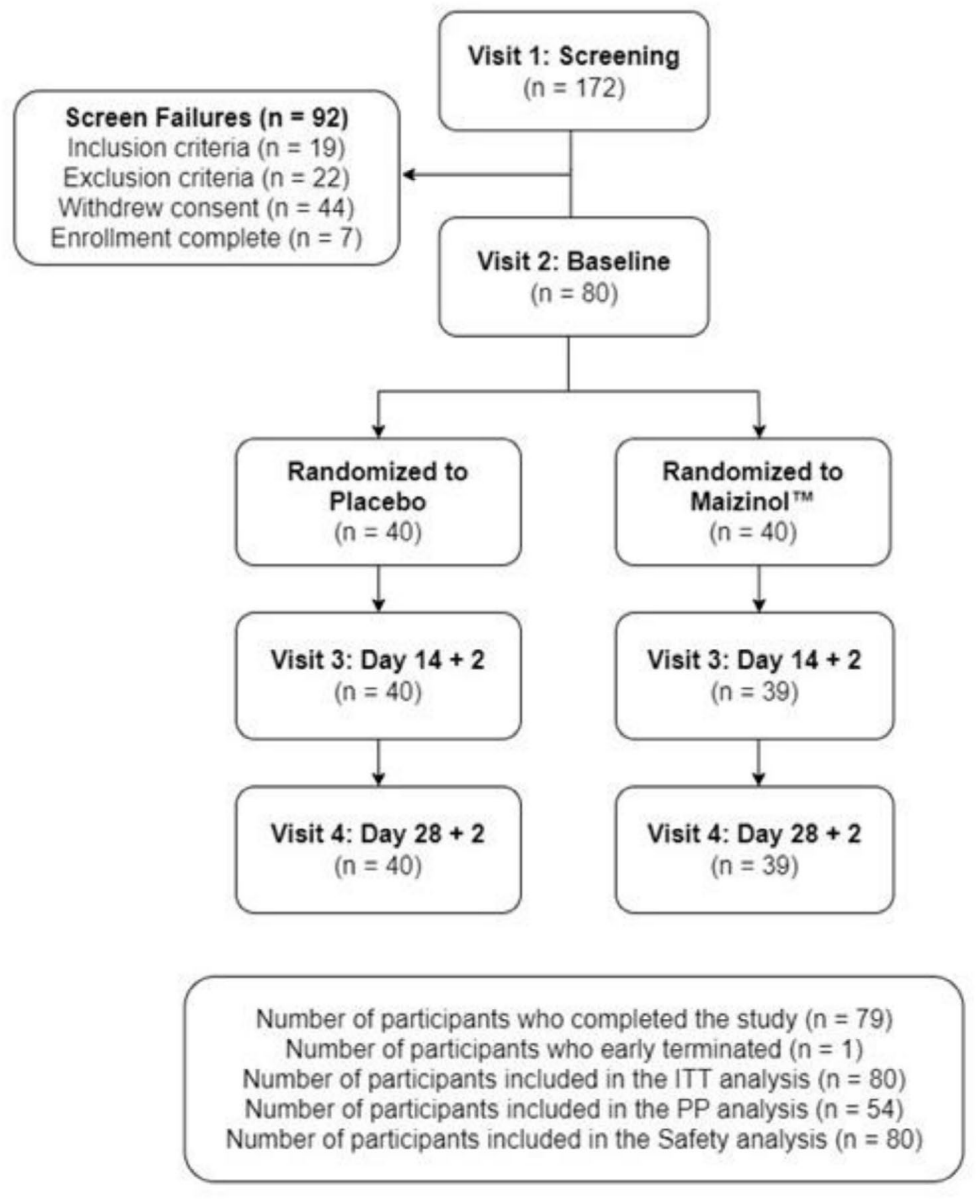
Participant disposition. CLE, corn leaf extract.

A total of 172 volunteers consented and were screened. Eighty eligible participants were enrolled in the study (*n* = 40 per group; Figure 2). Participants ranged from 18 to 65 years, and 70% identified as female, with no statistically significant differences in demographics or general health between groups (Table 1).

**TABLE 1:** Demographic and general health information of the study population (*n* = 80).

Variable	Placebo (*n* = 40)	CLE (*n* = 40)	*p*
Age (years)			
Mean ± SD	46.38 ± 12.21	45.92 ± 10.94	0.836
Median (Min to Max)	45.00 (18.00 to 62.00)	47.00 (22.00 to 65.00)	
Gender, *n* (%)			
Female	28 (70.00)	28 (70.00)	1.000
Male	12 (30.00)	12 (30.00)	
Race, *n* (%)			
Western European White	22 (55.00)	22 (55.00)	0.919^a^
Eastern European White	6 (15.00)	4 (10.00)	
Middle Eastern	3 (7.50)	2 (5.00)	
South American	2 (5.00)	2 (5.00)	
Southeast Asian	2 (5.00)	1 (2.50)	
Other	2 (5.00)	1 (2.50)	
Mixed	1 (2.50)	2 (5.00)	
African American or Black	1 (2.50)	2 (5.00)	
East Asian	1 (2.50)	0 (0.00)	
South Asian	0 (0.00)	2 (5.00)	
Central American	0 (0.00)	1 (2.50)	
Native or First Nations	0 (0.00)	1 (2.50)	
On a scale of 1–10, what is your current stress level?			
Mean ± SD	4.90 ± 2.05 (40)	4.80 ± 1.84 (40)	0.865
Median (Min to Max)	5.00 (1.00 to 8.00)	5.00 (1.00 to 8.00)	
Do you regularly exercise?			
Yes	22 (55.00%)	27 (67.50%)	0.251
No	18 (45.00%)	13 (32.50%)	
Does your stress affect your sleep?			
Yes	24 (60.00%)	28 (70.00%)	0.355
No	14 (35.00%)	9 (22.50%)	
Do you take any sleep aids/natural products/food/drink that help you to fall asleep?			
Yes	3 (7.50%)	3 (7.50%)	1.000^a^
No	37 (92.50%)	37 (92.50%)	
Do you consume caffeine‐containing products like tea, coffee, chocolate, and/or energy drinks regularly?			
Yes	35 (87.50%)	36 (90.00%)	1.000^a^
No	5 (12.50%)	4 (10.00%)	

*Note:* *P*‐values for continuous variables were calculated using Wilcoxon rank sum test. *P*‐values for categorical variables were calculated using Chi square test.

Abbreviations: Max, maximum; Min, minimum; *n*, number of participants; N/A, not applicable; SD, standard deviation.

^a^ *P*‐values for categorical variables were calculated using Fisher's exact test.

### 3.1 Objective Sleep Outcomes as Assessed by the Actigraphy With EEG/EOG

**FIGURE 3:**
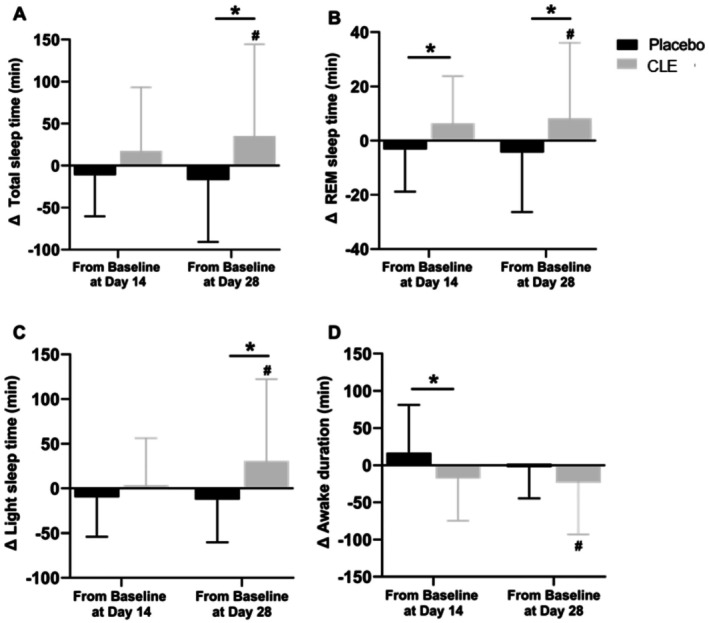
Change in objective sleep outcomes (A) total sleep time, (B) REM sleep time, (C) light sleep time, and (D) wake after sleep onset from baseline at Days 14 and 28. Data are mean ± standard deviation. CLE, corn leaf extract; REM, rapid‐eye movement. *p ≤ 0.05 compared to placebo; #*p* ≤ 0.05 for change from baseline.

Participants supplemented with 500 mg CLE 60 min before bedtime had significant increases in objective sleep measures of total sleep time (Δ = 35.16 min, *d* = 0.55, *p* = 0.033), REM sleep (Δ = 8.27 min, *d* = 0.50, *p* = 0.028), and light sleep time (Δ = 30.80 min, *d* = 0.59, *p* = 0.022) at Day 28 with a significant increase in REM sleep time (Δ = 6.40 min, *d* = 0.58, *p* = 0.042) and a decrease in awake duration (wake after sleep onset (WASO); Δ = −17.91 min, *d* = 0.58, *p* = 0.021) at Day 14 compared to placebo (Figure 3A–D). There was a significantly shorter WASO (81.74 vs. 129.45 min, *d* = 0.84, *p* = 0.001; 73.71 vs. 111.41 min, *d* = 0.67, *p* = 0.007) and higher sleep efficiency (80.96% vs. 71.39%, *d* = 1.01, *p* = 0.001; 81.03% vs. 74.04%, *d* = 0.59, *p* = 0.007) for the CLE group compared to the placebo group at Days 14 and 28, respectively (Table 2).

**TABLE 2:** Objective sleep outcomes as assessed by the actigraphy and EEG device from baseline at Days 14 and 28 (*n* = 80).

	Placebo (*n*)	CLE (*n*)	*p* ^e^
Deep sleep (min)			
Baseline (Day 0)			
Mean ± SD	84.58 ± 39.13 (33)^b^	89.96 ± 25.78 (36)^b^	0.382
Day 14			
Mean ± SD	80.45 ± 40.75 (33)^c^	97.45 ± 28.96 (33)^c^	0.134
*p* ^a^	0.648	0.063	
Day 28			
Mean ± SD	85.70 ± 38.65 (30)^d^	93.36 ± 26.13 (29)^d^	0.208
*p* ^a^	0.919	0.334	
Sleep efficiency (%)			
Baseline (Day 0)			
Mean ± SD	74.25 ± 11.63 (33)^b^	77.53 ± 9.10 (36)^b^	0.289
Day 14			
Mean ± SD	71.39 ± 10.91 (33)^c^	80.96 ± 7.77 (33)^c^	0.001
*p* ^a^	0.147	0.072	
Day 28			
Mean ± SD	74.04 ± 13.46 (30)^d^	81.03 ± 9.63 (29)^d^	0.007
*p* ^a^	0.702	0.063	
Sleep latency (min)			
Baseline (Day 0)			
Mean ± SD	51.70 ± 42.69 (33)^b^	32.98 ± 19.80 (36)^b^	0.053
Day 14			
Mean ± SD	56.39 ± 53.71 (33)^c^	33.70 ± 25.80 (33)^c^	0.008
*p* ^a^	0.224	0.980	
Day 28			
Mean ± SD	48.84 ± 39.94 (30)^d^	29.86 ± 24.16 (29)^d^	0.054
*p* ^a^	0.605	0.518	
Wake after sleep onset (WASO; min)			
Baseline (Day 0)			
Mean ± SD	114.80 ± 60.59 (33)^b^	95.94 ± 51.77 (36)^b^	0.240
Day 14			
Mean ± SD	129.45 ± 67.90 (33)^c^	81.74 ± 42.49 (33)^c^	0.001
*p* ^a^	0.118	0.086	
Day 28			
Mean ± SD	111.41 ± 67.92 (30)^d^	73.71 ± 39.79 (29)^d^	0.007
*p* ^a^	0.882	0.017	
Number of awakenings > 3 min (per hour)			
Baseline (Day 0)			
Mean ± SD	0.59 ± 0.49 (33)^b^	0.64 ± 0.69 (36)^b^	0.646
Day 14			
Mean ± SD	0.71 ± 0.57 (33)^c^	0.42 ± 0.34 (33)^c^	0.022
*p* ^a^	0.231	0.013	
Day 28			
Mean ± SD	0.49 ± 0.42 (30)^d^	0.36 ± 0.28 (29)^d^	0.182
*p* ^a^	0.571	0.004	

*Note:* *P*‐values were calculated using Linear Mixed Model analysis.

Abbreviations: *n*, number; SD, standard deviation.

^a^ *P*‐value for differences from baseline.

^b^ *n* = 11 missing baseline data.

^c^ *n* = 14 missing Day 14 data.

^d^ *n* = 21 missing Day 28 data.

^e^ *P*‐value for differences between groups.

There was a statistically significant reduction in the number of awakenings greater than 3 min for CLE compared to placebo at Day 14 (Table 2). At Day 14, participants in the CLE group had a significantly shorter sleep onset latency (33.70 vs. 56.39 min, *d* = 0.54, *p* = 0.008), which approached statistical significance at Day 28 (29.86 vs. 48.84 min, *d* = 0.57, *p* = 0.054).

Post hoc analysis of NREM sleep time showed a significant increase of 35.7 min at Day 28 for the CLE group compared to a decrease of −10.6 min for the Placebo group. There was also an increase of 9.4 min and a decrease of −8.0 min for the CLE and Placebo groups, respectively, at Day 14 (*p* = 0.193).

Participants supplemented with CLE had an increase of 5.88 ± 22.77 min in deep sleep time at Day 14, which approached statistical significance (*p* = 0.062) whereas those on placebo had an increase of 1.80 ± 17.87 min (*p* = 0.648) (Table 2).

### 3.2 Subjective Sleep Outcomes as Assessed by the PSQI

There were statistically significant decreases in subjective sleep domains, including the PSQI global score, sleep quality, sleep latency, sleep duration, and daytime dysfunction, from baseline at Days 14 and 28 for the CLE group (Table 3). The placebo group reported statistically significant decreases in PSQI global score, sleep quality, sleep duration, sleep disturbances, and daytime dysfunction from baseline at Days 14 and 28 (Table 3). There were no statistically significant differences in PSQI measures between groups.

**TABLE 3:** Subjective sleep measures as assessed by the PSQI global score and component scores from baseline at Days 14 and 28 (*n* = 80).

	Placebo (*n*)	CLE (*n*)	*p* ^a^
PSQI global score			
Baseline (Day 0)			
Mean ± SD	9.75 ± 2.20 (40)	10.03 ± 2.41 (40)	0.596
Change from baseline at day 14			
Mean ± SD	−1.10 ± 2.38 (40)	−1.23 ± 2.17 (39)^c^	0.863
*P* ^b^	< 0.001	< 0.001	
Change from baseline at day 28			
Mean ± SD	−1.43 ± 2.37 (40)	−1.74 ± 2.26 (39)^c^	0.589
*P* ^b^	< 0.001	< 0.001	
Subjective sleep quality			
Baseline (Day 0)			
Mean ± SD	1.62 ± 0.59 (40)	1.68 ± 0.62 (40)	0.711
Change from baseline at day 14			
Mean ± SD	−0.32 ± 0.62 (40)	−0.28 ± 0.69 (39)^c^	0.714
*P* ^b^	< 0.001	0.010	
Change from baseline at day 28			
Mean ± SD	−0.40 ± 0.63 (40)	−0.41 ± 0.72 (39)^c^	0.996
*P* ^b^	< 0.001	< 0.001	
Sleep latency			
Baseline (Day 0)			
Mean ± SD	2.17 ± 0.93 (40)	2.28 ± 0.64 (40)	0.577
Change from baseline at day 14			
Mean ± SD	−0.17 ± 0.84 (40)	−0.49 ± 0.85 (39)^c^	0.126
*P* ^b^	0.200	< 0.001	
Change from baseline at day 28			
Mean ± SD	−0.35 ± 0.92 (40)	−0.59 ± 0.79 (39)^c^	0.252
*P* ^b^	0.010	< 0.001	
Sleep duration			
Baseline (Day 0)			
Mean ± SD	1.07 ± 1.02 (40)	1.32 ± 0.92 (40)	0.253
Change from baseline at day 14			
Mean ± SD	−0.38 ± 0.84 (40)	−0.49 ± 0.72 (39)^c^	0.537
*P* ^b^	< 0.001	< 0.001	
Change from baseline at day 28			
Mean ± SD	−0.42 ± 0.81 (40)	−0.56 ± 0.82 (39)^c^	0.461
*P* ^b^	< 0.001	< 0.001	
Sleep efficiency			
Baseline (Day 0)			
Mean ± SD	2.40 ± 1.17 (40)	2.22 ± 1.31 (40)	0.531
Change from baseline at day 14			
Mean ± SD	0.28 ± 1.26 (40)	0.31 ± 1.36 (39)^c^	0.948
*P* ^b^	0.190	0.160	
Change from baseline at day 28			
Mean ± SD	0.28 ± 1.15 (40)	0.38 ± 1.04 (39)^c^	0.687
*P* ^b^	0.120	0.040	
Sleep disturbances			
Baseline (day 0)			
Mean ± SD	1.40 ± 0.50 (40)	1.30 ± 0.46 (40)	0.355
Change from baseline at day 14			
Mean ± SD	−0.20 ± 0.52 (40)	−0.05 ± 0.46 (39)^c^	0.167
*P* ^b^	0.010	0.540	
Change from baseline at day 28			
Mean ± SD	−0.25 ± 0.59 (40)	−0.13 ± 0.41 (39)^c^	0.270
*P* ^b^	< 0.001	0.130	
Daytime dysfunction			
Baseline (day 0)			
Mean ± SD	1.07 ± 0.69 (40)	1.23 ± 0.66 (40)	0.325
Change from baseline at day 14			
Mean ± SD	−0.30 ± 0.69 (40)	−0.31 ± 0.66 (39)^c^	0.959
*P* ^b^	0.010	0.010	
Change from baseline at day 28			
Mean ± SD	−0.38 ± 0.74 (40)	−0.51 ± 0.64 (39)^c^	0.443
*P* ^b^	< 0.001	< 0.001	

*Note:* *P*‐values were calculated using Linear Mixed Model analysis.

Abbreviations: n, number; SD, standard deviation.

^a^ *P*‐value for differences between groups.

^b^ *P*‐value for differences from baseline.

^c^ *n* = 1 early termination.

### 3.3 Sleep‐Related Blood Markers

Participants supplemented with CLE had respective decreases of −26.8 pmol/L (*p* = 0.002) and −13.5 pmol/L (*p* = 0.270) in melatonin from baseline at Day 14 and 28, whereas those on placebo had decreases of −9.4 pmol/L (*p* = 0.282) and −25.9 pmol/L (*p* = 0.047), respectively. There were no statistically significant differences in serotonin or GABA (data not shown).

### 3.4 Safety Outcomes

One post‐emergent AE of diarrhea in the placebo group was classified as mild in intensity and “possibly” related to the study product. There were no AEs related to vital signs. All post‐emergent AEs were resolved by the end of the study period or upon subsequent follow‐up. All out‐of‐range hematology values were deemed not clinically relevant as assessed by the QI, except for one participant in the placebo group with clinically relevant WBC and absolute neutrophils at Day 28. Blood work was repeated, and WBC was no longer clinically relevant, and absolute neutrophils returned to normal. All out‐of‐range clinical chemistry values were deemed not clinically relevant as assessed by the QI.

## 4 Discussion

This randomized, triple‐blind, placebo‐controlled clinical trial demonstrated that supplementation with CLE significantly improved sleep latency, WASO, sleep efficiency, and sleep interruptions in a population of healthy adults with self‐reported difficulties falling and/or staying asleep. Further, CLE supplementation resulted in significant improvements in REM sleep time, as assessed by actigraphy and EEG, a component of the gold‐standard polysomnography. The majority of structured dreaming occurs during the REM sleep stage and has been proposed to play a role in various biological functions, including memory formation and consolidation (Peever and Fuller 2017), and emotional regulation (Blumberg et al. 2020). Additionally, a decrease in REM sleep has been inversely associated with all‐cause and cardiovascular mortality (Leary et al. 2020). One of the key considerations in developing a sleep aid product is preserving natural sleep architecture. In this clinical study, supplementation with CLE increased REM sleep time in proportion to total sleep time, without disrupting sleep architecture. This is an important distinction, as GABAergic agents like benzodiazepines often interfere with sleep architecture, which can lead to cognitive impairment the following day.

In addition to the significant improvements observed for REM sleep, TST significantly increased after CLE supplementation compared to placebo. The increase in TST suggests that participants in the CLE group experienced adequate sleep, allowing them to spend sufficient time in both REM and NREM stages. This likely benefited the psychological and biological functions associated with these phases of sleep. Increases in TST support findings from a previous clinical trial where healthy adults were supplemented with the same IP (CLE) (Talbott et al. 2023). The current study's findings corresponded with improvements in sleep efficiency and awake duration, further benefiting the studied population, as deficits in these sleep variables have been associated with hypertension, cardiovascular disease, and cognitive impairment (Ikeda et al. 2022; Yan et al. 2021). Post hoc analysis also showed significant improvements after 28 days of CLE supplementation in NREM sleep compared to placebo, supporting findings from a rodent study with the same IP (Kim et al. 2024). This is relevant for the studied population because of reported difficulties falling and staying asleep, as NREM is an important sleep type characterized by rest and restoration (Sleep Foundation 2022). Significant increases in NREM sleep were observed after supplementation with melatonin in adults with unselected neuropsychiatric sleep disorders and reduced REM sleep duration (Kunz et al. 2004).

Although the mean increase in deep sleep time at both study timepoints was greater after CLE supplementation in comparison to placebo, these differences did not reach statistical significance. The improvements in deep sleep time are consistent with a previous study that showed significant increases in deep sleep after supplementation with the same IP (CLE) (Talbott et al. 2023). The lack of statistically significant differences in the current versus previous study may be explained by differing methods to assess sleep, baseline deep sleep time, and inter‐individual variability. Participants in the current study had a mean deep sleep time at baseline of 90.0 min and 84.6 min for the CLE and placebo groups, respectively, compared to 67.6 min and 57.9 min for the CLE and placebo groups in the previous study. With an approximately 25%–32% higher baseline deep sleep time for the participants in the current trial, there may have been less room for meaningful improvement. It is unclear if the use of differing methods to assess sleep led to the differences in baseline deep sleep and observed greater inter‐individual variability. Given the inconsistencies in study findings, future studies utilizing the same objective sleep device are warranted to enable direct comparison between studies.

In addition to improvements in objective sleep measures, participants supplemented with CLE reported significant improvements from baseline in overall sleep quality, as assessed by the PSQI global score. Although significant improvements were also observed for the placebo, the magnitude of improvement was greater with CLE supplementation. These findings are important as poor subjective sleep quality has been associated with cardiovascular disease risk factors and coronary heart disease (Kwok et al. 2018; Valenzuela et al. 2022). However, there were no significant differences between groups, despite those found from the objective EEG and actigraphy device. Similar findings have been reported previously in an adult population with small sleep disorders but who were not patients, as described by the authors (Yamatsu et al. 2016), and in adults with unselected neuropsychiatric sleep disorders (Kunz et al. 2004). The actigraphy device is commonly used in sleep research and previously validated against the gold standard, polysomnography (Ansbjerg et al. 2023; Conley et al. 2019; Cousins et al. 2019; de Souza et al. 2003; Kumagai et al. 2022; Misaka et al. 2023; Morgenthaler et al. 2007; Perini et al. 2023; Tantrakul et al. 2023), with actigraphy and EEG showing high agreement with polysomnography (Fietze et al. 2015), whereas the PSQI is a validated self‐report tool used to assess sleep quality (Buysse et al. 1989). Although the subjective sleep outcomes were not statistically significantly different between groups, there was agreement in the directionality of change for some comparable variables. Inconsistency in findings may be due to inherent limitations of self‐reported assessments, particularly related to memory recall (Fabbri et al. 2021), or the study population whereas the lack of statistically significant differences between groups may be due to the placebo effect which relates to psychological expectations that lead to perceived improvements (Lee et al. 2021; Patterson et al. 2024; Talbott et al. 2023).

The improvements in objective and subjective sleep parameters in the CLE group were observed despite no significant changes in blood levels of serotonin or GABA. Although serotonin levels in the current study are comparable to previous literature (Hirowatari et al. 2011), GABA levels appear to be lower than previously reported in healthy adults (Lyssikatos et al. 2023; Yamatsu et al. 2016). As GABA may be impaired in individuals with sleep disorders (Winkelman et al. 2008), this may explain the lower levels of GABA in the studied population because of their self‐reported sleep difficulties despite being otherwise healthy. Another relevant blood marker, melatonin, significantly decreased from baseline at Day 14, but this decrease was not statistically significant after 28 days of supplementation. CLE has been proposed to regulate the synthesis of melatonin and serotonin via 6‐MBOA. Given the circadian rhythm of melatonin production (Kennaway and Wright 2002), a single diurnal measurement may not have provided a complete understanding of the effects of CLE on this sleep hormone and contributed to the stated results. Given the proposed mechanism of action and timing of CLE supplementation, future studies should consider measuring melatonin, GABA and serotonin levels near the time of administration and multiple timepoints throughout the day and night, or quantify the total metabolites of melatonin in urine to better understand the effects of CLE on these markers.

Supplementation with CLE was found to be safe and well‐tolerated in this population of healthy adults with self‐reported difficulty falling or staying asleep, further supporting the safety profile of CLE (Kalman et al. 2015; Talbott et al. 2023). Current pharmacologic sleep aids are associated with AEs such as confusion, diarrhea, dizziness, memory impairment, weakness, and may lead to dependency (Lie et al. 2015; Proctor and Bianchi 2012) which were not reported with CLE 500 mg oral supplementation. In fact, there was only one AE classified as “possibly” related to the product, which was reported in the placebo group. Confirmation of the safety and tolerability of CLE is an important step given the AEs associated with current pharmacologic sleep aids and the need for safer alternatives.

Findings from the current study need to be interpreted in the context of study limitations. The study duration was only 28 days, and although findings suggest improvements in sleep outcomes may occur shortly after CLE supplementation, future longer studies are warranted to understand long‐term efficacy and safety. Further, demographics of the studied population need to be considered when interpreting results, as generalizability may be limited because of 65%–70% of participants being European White and female. Future research exploring CLE supplementation in more diverse populations is needed.

## 5 Conclusions

This randomized, triple‐blind, placebo‐controlled clinical trial demonstrated that supplementation with 500 mg of CLE for 28 days significantly improved objective REM sleep, NREM, total sleep time, and light sleep time compared to placebo in a population with self‐reported difficulties falling or staying asleep. These findings were supported by improvements in objective sleep latency, awake duration, higher sleep efficiency, and subjective measures of sleep quality. Taken together, the findings suggest that CLE may play a role in improving various aspects of sleep, relevant for the maintenance of health and risk reduction of chronic disease. Importantly, CLE supplementation was safe and well‐tolerated, which is relevant given the safety concerns of prescription sleep aids. Studies of longer duration with more diverse study populations will further our understanding of CLE supplementation for sleep.

## Author Contributions

**Katarina M. Doma:** visualization (equal), writing – original draft (equal), writing – review and editing (equal). **Abdelrahman Zamzam:** formal analysis (lead). **David C. Crowley:** data curation (lead), investigation (lead), methodology (equal). **Najla Guthrie:** conceptualization (equal), funding acquisition (lead), supervision (equal). **Erin D. Lewis:** conceptualization (equal), methodology (equal), supervision (equal), visualization (equal), writing – original draft (equal), writing – review and editing (equal).

## Funding

This work was supported by Unigen Inc.

## Ethics Statement

Notice of authorization was granted on October 31, 2022, by the Natural and Non‐Prescription Health Product Directorate, Health Canada, and unconditional approval was granted on November 18, 2022, by the Institutional Review Board (Advarra, Aurora, Ontario, Canada; Pro00067855).

## Consent

Informed consent was obtained from all participants during the screening visit prior to performing study procedures.

## Conflicts of Interest

The authors declare no conflicts of interest.

## Data Availability

The data that support the findings of this study are available from the corresponding author upon reasonable request.

## References

[fsn371285-bib-0001] Anderson, K. D. , R. J. Nachman , and F. W. Turek . 1988. “Effects of Melatonin and 6‐Methoxybenzoxazolinone on Photoperiodic Control of Testis Size in Adult Male Golden Hamsters.” Journal of Pineal Research 5, no. 4: 351–365. 10.1111/j.1600-079X.1988.tb00884.x. 3210136

[fsn371285-bib-0002] Ansbjerg, M. B. , H. Sandahl , L. Baandrup , P. Jennum , and J. Carlsson . 2023. “Sleep Impairments in Refugees Diagnosed With Post‐Traumatic Stress Disorder: A Polysomnographic and Self‐Report Study.” European Journal of Psychotraumatology 14, no. 1: 2185943. 10.1080/20008066.2023.2185943. 36971225 PMC10044313

[fsn371285-bib-0003] Blumberg, M. S. , J. A. Lesku , P.‐A. Libourel , M. H. Schmidt , and N. C. Rattenborg . 2020. “What Is REM Sleep?” Current Biology 30, no. 1: R38–R49. 10.1016/j.cub.2019.11.045. 31910377 PMC6986372

[fsn371285-bib-0004] Buysse, D. J. , C. F. Reynolds , T. H. Monk , S. R. Berman , and D. J. Kupfer . 1989. “The Pittsburgh Sleep Quality Index: A New Instrument for Psychiatric Practice and Research.” Psychiatry Research 28, no. 2: 193–213. 10.1016/0165-1781(89)90047-4. 2748771

[fsn371285-bib-0005] Cohen, J. 2013. Statistical Power Analysis for the Behavioral Sciences. 2nd ed. Routledge. 10.4324/9780203771587.

[fsn371285-bib-0006] Conley, S. , A. Knies , J. Batten , et al. 2019. “Agreement Between Actigraphic and Polysomnographic Measures of Sleep in Adults With and Without Chronic Conditions: A Systematic Review and Meta‐Analysis.” Sleep Medicine Reviews 46: 151–160. 10.1016/j.smrv.2019.05.001. 31154154 PMC6594867

[fsn371285-bib-0007] Cousins, J. N. , E. Van Rijn , J. L. Ong , and M. W. L. Chee . 2019. “A Split Sleep Schedule Rescues Short‐Term Topographical Memory After Multiple Nights of Sleep Restriction.” Sleep 42, no. 4: zsz018. 10.1093/sleep/zsz018. 30715485 PMC6448285

[fsn371285-bib-0008] de Souza, L. , A. A. Benedito‐Silva , M. L. N. Pires , D. Poyares , S. Tufik , and H. M. Calil . 2003. “Further Validation of Actigraphy for Sleep Studies.” Sleep 26, no. 1: 81–85. 10.1093/sleep/26.1.81. 12627737

[fsn371285-bib-0009] Fabbri, M. , A. Beracci , M. Martoni , D. Meneo , L. Tonetti , and V. Natale . 2021. “Measuring Subjective Sleep Quality: A Review.” International Journal of Environmental Research and Public Health 18, no. 3: 1082. 10.3390/ijerph18031082. 33530453 PMC7908437

[fsn371285-bib-0010] Fietze, I. , T. Penzel , M. Partinen , et al. 2015. “Actigraphy Combined With EEG Compared to Polysomnography in Sleep Apnea Patients.” Physiological Measurement 36, no. 3: 385–396. 10.1088/0967-3334/36/3/385. 25651914

[fsn371285-bib-0011] Gobbi, G. , and S. Comai . 2019. “Differential Function of Melatonin MT_1_ and MT_2_ Receptors in REM and NREM Sleep.” Frontiers in Endocrinology 10: 87. 10.3389/fendo.2019.00087. 30881340 PMC6407453

[fsn371285-bib-0012] Hirowatari, Y. , K. Hara , Y. Shimura , and H. Takahashi . 2011. “Serotonin Levels in Platelet‐Poor Plasma and Whole Blood From Healthy Subjects: Relationship With Lipid Markers and Coronary Heart Disease Risk Score.” Journal of Atherosclerosis and Thrombosis 18, no. 10: 874–882. 10.5551/jat.8995. 21712614

[fsn371285-bib-0013] Hou, X. , J. Hu , E. Wang , et al. 2023. “Self‐Reported Sleep Disturbance Is an Independent Predictor of All‐Cause Mortality and Respiratory Disease Mortality in US Adults: A Population‐Based Prospective Cohort Study.” International Journal of Public Health 68: 1605538. 10.3389/ijph.2023.1605538. 36865999 PMC9971003

[fsn371285-bib-0014] Ikeda, Y. , E. Morita , K. Muroi , et al. 2022. “Relationships Between Sleep Efficiency and Lifestyle Evaluated by Objective Sleep Assessment: SLeep Epidemiology Project at University of Tsukuba [Nagoya University Graduate School of Medicine, School of Medicine].” Nagoya Journal of Medical Science 84, no. 3: 554–569. https://nagoya.repo.nii.ac.jp/records/2003748. 36237889 10.18999/nagjms.84.3.554 PMC9529619

[fsn371285-bib-0015] Kalman, D. S. , S. Feldman , R. R. Vazquez , and D. R. Krieger . 2015. “A Prospective Randomized Double‐Blind Study Evaluating UP165 and S‐Adenosyl‐l‐Methionine on Depression, Anxiety and Psychological Well‐Being.” Food 4, no. 2: 130–139. 10.3390/foods4020130. PMC5302331 28231193

[fsn371285-bib-0016] Kennaway, D. J. , and H. Wright . 2002. “Melatonin and Circadian Rhythms.” Current Topics in Medicinal Chemistry 2: 199–209. https://www.eurekaselect.com/article/26306. 11899101 10.2174/1568026023394380

[fsn371285-bib-0017] Kim, H. J. , H. Kim , Y. J. Kim , et al. 2024. “UP165, Standardized Corn Leaf Extract and Its Active Component 6‐Methoxybenzoxazolinone Induce Non‐Rapid Eye Movement Sleep Through Melatonergic and GABAergic Mechanisms.” Food Bioscience 61: 104584. 10.1016/j.fbio.2024.104584.

[fsn371285-bib-0018] Kumagai, H. , H. Sawatari , T. Hoshino , et al. 2022. “Effects of Continuous Positive Airway Pressure Therapy on Nocturnal Blood Pressure Fluctuation Patterns in Patients With Obstructive Sleep Apnea.” International Journal of Environmental Research and Public Health 19, no. 16: 9906. 10.3390/ijerph19169906. 36011538 PMC9407792

[fsn371285-bib-0019] Kunz, D. , R. Mahlberg , C. Müller , A. Tilmann , and F. Bes . 2004. “Melatonin in Patients With Reduced REM Sleep Duration: Two Randomized Controlled Trials.” Journal of Clinical Endocrinology & Metabolism 89, no. 1: 128–134. 10.1210/jc.2002-021057. 14715839

[fsn371285-bib-0020] Kwok, C. S. , E. Kontopantelis , G. Kuligowski , et al. 2018. “Self‐Reported Sleep Duration and Quality and Cardiovascular Disease and Mortality: A Dose‐Response Meta‐Analysis.” Journal of the American Heart Association 7, no. 15: e008552. 10.1161/JAHA.118.008552. 30371228 PMC6201443

[fsn371285-bib-0021] Leary, E. B. , K. T. Watson , S. Ancoli‐Israel , et al. 2020. “Association of Rapid Eye Movement Sleep With Mortality in Middle‐Aged and Older Adults.” JAMA Neurology 77, no. 10: 1241–1251. 10.1001/jamaneurol.2020.2108. 32628261 PMC7550971

[fsn371285-bib-0022] Lee, H. J. , J. K. Hong , J.‐K. Kim , et al. 2021. “Effects of Probiotic NVP‐1704 on Mental Health and Sleep in Healthy Adults: An 8‐Week Randomized, Double‐Blind, Placebo‐Controlled Trial.” Nutrients 13, no. 8: 2660. 10.3390/nu13082660. 34444820 PMC8398773

[fsn371285-bib-0023] Lie, J. D. , K. N. Tu , D. D. Shen , and B. M. Wong . 2015. “Pharmacological Treatment of Insomnia.” Pharmacy and Therapeutics 40, no. 11: 759–771. 26609210 PMC4634348

[fsn371285-bib-0024] Lyssikatos, C. , Z. Wang , Z. Liu , S. J. Warden , L. Bonewald , and M. Brotto . 2023. “γ‐Aminobutyric Acids (GABA) and Serum GABA/AABA (G/A) Ratio as Potential Biomarkers of Physical Performance and Aging.” Scientific Reports 13, no. 1: 17083. 10.1038/s41598-023-41628-x. 37816783 PMC10564855

[fsn371285-bib-0044] Malhotra, R. K. , and A. Y. Avidan . 2013. “Sleep Stages and Scoring Technique.” In Atlas of Sleep Medicine, 77–99. Springer.

[fsn371285-bib-0025] Misaka, T. , A. Yoshihisa , T. Yokokawa , and Y. Takeishi . 2023. “Effects of Continuous Positive Airway Pressure on Very Short‐Term Blood Pressure Variability Associated With Sleep‐Disordered Breathing by Pulse Transit Time‐Based Blood Pressure Measurements.” Journal of Hypertension 41, no. 5: 733–740. 10.1097/HJH.0000000000003395. 36883467

[fsn371285-bib-0026] Morgenthaler, T. , C. Alessi , L. Friedman , et al. 2007. “Practice Parameters for the Use of Actigraphy in the Assessment of Sleep and Sleep Disorders: An Update for 2007.” Sleep 30, no. 4: 519–529. 10.1093/sleep/30.4.519. 17520797

[fsn371285-bib-0027] NCHS . 2022. “Products—Data Briefs—Number 436—June 2022.” 10.15620/cdc:117490.

[fsn371285-bib-0028] NCHS . 2023. “Products—Data Briefs—Number 462—January 2023.” 10.15620/cdc:123013.

[fsn371285-bib-0029] Patterson, E. , H. T. T. Tan , D. Groeger , et al. 2024. “Bifidobacterium Longum 1714 Improves Sleep Quality and Aspects of Well‐Being in Healthy Adults: A Randomized, Double‐Blind, Placebo‐Controlled Clinical Trial.” Scientific Reports 14, no. 1: 3725. 10.1038/s41598-024-53810-w. 38355674 PMC10866977

[fsn371285-bib-0030] Peever, J. , and P. M. Fuller . 2017. “The Biology of REM Sleep.” Current Biology 27, no. 22: R1237–R1248. 10.1016/j.cub.2017.10.026. 29161567

[fsn371285-bib-0031] Perini, F. , K. F. Wong , J. Lin , et al. 2023. “Mindfulness‐Based Therapy for Insomnia for Older Adults With Sleep Difficulties: A Randomized Clinical Trial.” Psychological Medicine 53, no. 3: 1038–1048. 10.1017/S0033291721002476. 34193328 PMC9975962

[fsn371285-bib-0032] Proctor, A. , and M. T. Bianchi . 2012. “Clinical Pharmacology in Sleep Medicine.” International Scholarly Research Notices 2012, no. 1: 914168. 10.5402/2012/914168.

[fsn371285-bib-0033] Salehinejad, M. A. , A. Azarkolah , E. Ghanavati , and M. A. Nitsche . 2022. “Circadian Disturbances, Sleep Difficulties and the COVID‐19 Pandemic.” Sleep Medicine 91: 246–252. 10.1016/j.sleep.2021.07.011. 34334305 PMC8277544

[fsn371285-bib-0045] Sleep Computing Committee and Japanese Society of Sleep Research . 2012. Learning Manual of PSG Chart. Japanese Society of Sleep Research.

[fsn371285-bib-0034] Sleep Foundation . 2020. Side Effects of Sleep Medication Sleep Foundation. https://www.sleepfoundation.org/sleep‐aids/side‐effects‐of‐sleeping‐pills.

[fsn371285-bib-0035] Sleep Foundation . 2022. “What Is NREM Sleep?” Sleep Foundation. https://www.sleepfoundation.org/stages‐of‐sleep/nrem‐sleep.

[fsn371285-bib-0036] Sweat, F. W. , and P. J. Berger . 1988. “Uterotropic 6‐Methoxybenzoxazolinone Is an Adrenergic Agonist and a Melatonin Analog.” Molecular and Cellular Endocrinology 57, no. 1: 131–138. 10.1016/0303-7207(88)90042-1. 2899525

[fsn371285-bib-0037] Talbott, S. M. , J. A. Talbott , L. Brownell , and M. Yimam . 2023. “UP165, A Standardized Corn Leaf Extract for Improving Sleep Quality and Mood State.” Journal of Medicinal Food 26, no. 1: 59–67. 10.1089/jmf.2021.0197. 36179066 PMC9889011

[fsn371285-bib-0038] Tantrakul, V. , A. Ingsathit , S. Liamsombut , et al. 2023. “Treatment of Obstructive Sleep Apnea in High Risk Pregnancy: A Multicenter Randomized Controlled Trial.” Respiratory Research 24, no. 1: 171. 10.1186/s12931-023-02445-y. 37370135 PMC10294320

[fsn371285-bib-0039] Valenzuela, P. L. , A. Santos‐Lozano , A. Torres‐Barrán , et al. 2022. “Poor Self‐Reported Sleep Is Associated With Risk Factors for Cardiovascular Disease: A Cross‐Sectional Analysis in Half a Million Adults.” European Journal of Clinical Investigation 52, no. 5: e13738. 10.1111/eci.13738. 34958676

[fsn371285-bib-0040] Winkelman, J. W. , O. M. Buxton , J. E. Jensen , et al. 2008. “Reduced Brain GABA in Primary Insomnia: Preliminary Data From 4T Proton Magnetic Resonance Spectroscopy (1H‐MRS).” Sleep 31, no. 11: 1499–1506. 10.1093/sleep/31.11.1499. 19014069 PMC2579978

[fsn371285-bib-0041] Yamatsu, A. , Y. Yamashita , T. Pandharipande , I. Maru , and M. Kim . 2016. “Effect of Oral γ‐Aminobutyric Acid (GABA) Administration on Sleep and Its Absorption in Humans.” Food Science and Biotechnology 25, no. 2: 547–551. 10.1007/s10068-016-0076-9. 30263304 PMC6049207

[fsn371285-bib-0042] Yan, B. , J. Yang , B. Zhao , Y. Fan , W. Wang , and X. Ma . 2021. “Objective Sleep Efficiency Predicts Cardiovascular Disease in a Community Population: The Sleep Heart Health Study.” Journal of the American Heart Association 10, no. 7: e016201. 10.1161/jaha.120.016201. 33719504 PMC8174351

[fsn371285-bib-0043] Yuwiler, A. , and W. D. Winters . 1985. “Effects of 6‐Methoxy‐2‐Benzoxazolinone on the Pineal Melatonin Generating System.” Journal of Pharmacology and Experimental Therapeutics 233, no. 1: 45–50. 10.1016/S0022-3565(25)21141-7. 3981461

